# STAT3, p-STAT3 and HIF-1α are associated with vasculogenic mimicry and impact on survival in gastric adenocarcinoma

**DOI:** 10.3892/ol.2014.2059

**Published:** 2014-04-11

**Authors:** YAN-YAN SONG, LI-DAN SUN, MIN-LI LIU, ZHONG-LIANG LIU, FEI CHEN, YING-ZHE ZHANG, YAN ZHENG, JIAN-PING ZHANG

**Affiliations:** 1 Department of Pathology, Shandong University School of Medicine, Jinan, Shandong 250012, P.R. China; 2 Department of Neurosurgery, Yidu Central Hospital, Weifang, Shandong 262500, P.R. China; 3 Department of Cardiology, Provincial Hospital Affiliated to Shandong University, Jinan, Shandong 250021, P.R. China; 4 Department of Surgery, Shandong University School of Medicine, Jinan, Shandong 250012, P.R. China; 5 Central Laboratory, Jinan Central Hospital Affiliated to Shandong University, Jinan, Shandong 250013, P.R. China; 6 Department of Pathology, Qilu Hospital, Shandong University, Jinan, Shandong 250012, P.R. China

**Keywords:** gastric adenocarcinoma, signal transducer and activator of transcription-3, phosphor-signal transducer and activator of transcription-3, hypoxia-inducible factor-1α, vasculogenic mimicry, prognosis

## Abstract

Vasculogenic mimicry (VM) formation is important for invasion and metastasis of tumor cells in gastric adenocarcinoma (GAC). The present study aimed to investigate the association between signal transducer and activator of transcription-3 (STAT3), phosphor-STAT3 (p-STAT3), hypoxia-inducible factor-1α (HIF-1α) and VM formation in GAC, and discuss their clinical significance and correlation with the prognosis of patients with GAC. The expression levels of STAT3, p-STAT3, HIF-1α and VM were assessed in 60 cases of patients with GAC and 20 cases of patients with gastritis on tissue microarrays by immunohistochemical methods. The expression levels of STAT3, p-STAT3, HIF-1α and VM were higher in patients with GAC (particularly in poorly differentiated GAC) than in those with gastritis (P<0.05). The expression levels of STAT3, p-STAT3 and HIF-1α were higher in VM tissues compared with non-VM tissues (P<0.05). Positive correlations existed between STAT3, p-STAT3, HIF-1α and VM expression (P<0.05). The expression levels of STAT3, p-STAT3 and HIF-1α, VM, status of lymph node metastasis and tumor differentiation degree were associated with the overall survival time of patients with GAC (P<0.05). However, only p-STAT3 and VM expression were identified as the independent risk factors of GAC OS when analyzed with multivariate analysis. p-STAT3 and VM play a significant role in indicating the prognosis of patients with GAC. STAT3 activation may play a positive role in VM formation of GAC by the STAT3-p-STAT3-HIF-1α-VM effect axis.

## Introduction

A blood supply is essential for the growth and hematogenous metastasis of tumors. Maniotis *et al* (1) previously reported an angiogenesis-independent pathway known as vasculogenic mimicry (VM). This pathway is a novel phenomenon in which highly aggressive human melanoma cells imitate endothelial cells and form vascular channel-like structures to convey blood plasma and red blood cells without the involvement of endothelial cells. Periodic acid-Schiff (PAS)-positive patterns identify VM channels. Subsequently, VM was identified in lung cancer, hepatocellular carcinoma, gallbladder carcinoma, gastric adenocarcinoma (GAC) and other types of cancer (2–5). A study by Li *et al* (5) described the expression of VM in GAC, particularly in poorly differentiated GAC. VM may play an extremely significant role in the biological behavior of multiple tumors (2–6). However, establishing the detailed mechanism of VM formation is required.

It has been reported that positive expression of hypoxia-inducible factor-1α (HIF-1α) is associated with the formation of VM in primary gallbladder, non-small cell lung cancer and hepatocellular carcinoma (2–4). STAT3 modulates the stability and activity of HIF-1α, and activated STAT3 increases the HIF-1α protein level by increasing HIF-1α stability through blocking HIF-1α degradation and accelerating its *de novo* synthesis (7). Pawlus *et al* (8) found that STAT3 exhibited specific binding to the promoters of HIF1 or HIF2 target genes respectively, even when overexpressed, and STAT3 interacted with HIF-1α to activate HIF1 target gene promoters. Taking into consideration the aforementioned details, STAT3 activation is possibly associated with VM formation. Therefore, investigating the association between STAT3 and VM formation in GAC is worthwhile to learn more about tumor development, invasion and metastasis.

In the present study, the expression levels of STAT3, p-STAT3, HIF-1α and VM were explored simultaneously for the first time. Firstly, the existence of VM in GAC was confirmed by a cluster of differentiation 31 (CD31)/PAS double-staining method. Subsequently, combining VM existence with the expression levels of STAT3, p-STAT3 and HIF-1α, the association was assessed between them and the possible formation mechanism of VM was investigated. Additionally, prognosis was assessed by Kaplan-Meier survival analysis for univariate analysis and by Cox proportional hazards model for multivariate analysis.

## Materials and methods

### Subjects

A total of 80 cases of paraffin-embedded specimens were collected in the Department of Pathology at the Qilu Hospital of Shandong University (Jinan, China). These cases included 60 GAC specimens (46 male and 14 female patients; median age, 60.0 years) and 20 gastritis specimens (11 male and 9 female patients; median age, 56.2 years). Primary gastric cancer in these patients was diagnosed and treated at the Qilu Hospital between July 2005 and December 2006. The patients with GAC had well-documented clinical histories and follow-up information. None of the patients underwent preoperative chemotherapy and/or radiation therapy. The follow-up time ranged between 6 and 72 months until July 2012, although the follow-up data of one case was lost. Overall survival (OS) time was defined as the interval between the dates of surgery and mortality. The gastritis cases were derived from gastritis biopsy specimens. All the cases were reviewed by two highly qualified pathologists. The study was approved by the ethics committee of Shandong University School of Medicine (Jinan, China) and written informed consent was obtained from the patients or their family.

### Construction of the tissue microarray

A tissue microarray instrument (HT-1 type; Hengtai Technology Development Co., Ltd., Chaoyang, China) was used to construct a blank receptor wax block of six rows and seven columns. Marked and collected tissues from the paraffin-embedded specimens were inserted into the holes of the receptor wax block. From each case, two specimens were acquired to overcome the loss of tissue. The first two holes on the first line were filled with ash, which served as a ‘blank’ specimen-positioning reference. Each receptor wax block accommodated 40 specimens, which represented a total of 20 cases. The GAC specimens were built into the three tissue microarrays. Each was subjected to repeated wax melting at 56°C to become a whole specimen. The tissue microarrays and gastritis tissue specimens were sectioned into 4-μm-thick slices that served as a continuous backup source.

### Immunohistochemical staining

The slices were dewaxed in xylene and then rehydrated through a graded series of alcohols. For antigen retrieval, the slides were heated in 10 mmol/l EDTA buffer (pH 8.0). Subsequent to washing with phosphate-buffered saline (PBS) three times, the endogenous peroxidase activity was blocked by 3% hydrogen peroxidase for 10 min of incubation at room temperature. Following washing with PBS again, the sections were incubated with polyclonal rabbit anti-human STAT3 (bs-1141R; Bioss, Inc., Beijing, China), polyclonal rabbit anti-phospho-STAT3 (bs-3429R; Bioss, Inc.) and monoclonal rabbit anti-human HIF-1α (ZA-0552; ZSGB-BIO, Beijing, China) primary antibodies at 4°C overnight separately. The slides were washed with PBS and incubated with biotinylated horseradish peroxidase-conjugated secondary antibody, polyclonal goat anti-rabbit immunoglobulin G (PV-6001; ZSGB-BIO), at room temperature for 30 min. Subsequent to washing, the slides were colored with 3,3-diaminobenzidine and counterstained with hematoxylin (9). VM was obtained by CD31/PAS double-staining, and monoclonal rabbit anti-human CD31 (ZA-0568; ZSGB-BIO) was colored with 3,3-diaminobenzidine(ZLI-9017; ZSGB-BIO). Then, the slides were placed in 10 mg/ml periodic acid buffer (P0430-25G; Sigma-Aldrich, Carlsbad, CA, USA) for 10 mins. Following washing with water, the slides were colored with Schiff (3952016; Sigma-Aldrich) for 15 min. Following washing with water, the slides were stained with hematoxylin (ZLI-9609; ZSGB-BIO).

### Immunohistochemical analysis

A positive result of immunohistochemical staining is characterized by the existence of yellow-to-brown granules. The positive staining of STAT3 was mainly located in the cytoplasm and partly in the nuclei, while the positive staining of p-STAT3 and HIF-1α was mainly located in the nuclei and partly in the cytoplasm. There were two factors that determined the final outcomes: The staining intensity observed under microscope (BX53, OLympus, Tokyo, Japan) and the proportion of positive cells estimated in an average of 100 cells counted in 10 high-magnification fields. The staining intensity was subjected to the following numerical scoring: Specimens were colorless, 0 points; pale yellow, 1 point; yellow, 2 points; or brown, 3 points. The proportion of positive cells was scored as follows: The number of positive cells was <5%, 0 points; 5–25%, 1 point; 26–50%, 2 points; 51–75%, 3 points; and >75%, 4 points. Immunostaining was considered positive when the product of the two types of scores was multiplied and was ≥4 (10). VM was identified in GAC tissues by CD31/PAS double-staining. VM, characterized by CD31-negative/PAS-positive vascular-like patterns and the presence of red blood cells, was formed by GAC cells, while typical blood vessels showed CD31-positive/PAS-negative in their vascular wall. All sections were scored blindly by two independent observers.

### Statistical analysis

The statistical analysis was performed with the SPSS Graduate Park 19.0 software (SPSS, Inc., Chicago, IL, USA). The count column was analyzed by the χ^2^ test. For the correlation analysis of STAT3, p-STAT3, HIF-1α and VM expression, Spearman's rank correlation test was applied; whereas for the survival analysis, the Kaplan-Meier method and Cox regression analysis were applied. P<0.05 was considered to indicate a statistically significant difference.

## Results

### STAT3, p-STAT3, HIF-1α and VM expression in GAC and gastritis tissues

**Figure 1:**
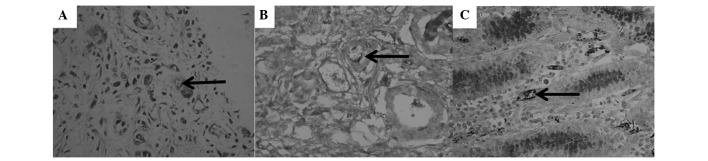
Expression of VM in GAC and gastritis tissues (magnification, ×400). (A) VM in GAC tissue (arrow, hematoxylin and eosin). (B) VM in GAC tissue (arrow, CD31/PAS). (C) VM-negative expression in gastritis tissue (arrow indicates typical blood vessel, CD31/PAS). VM, vasculogenic mimicry; GAC, gastric adenocarcinoma; CD, cluster of differentiation; PAS, periodic acid-Schiff.

**Figure 2:**
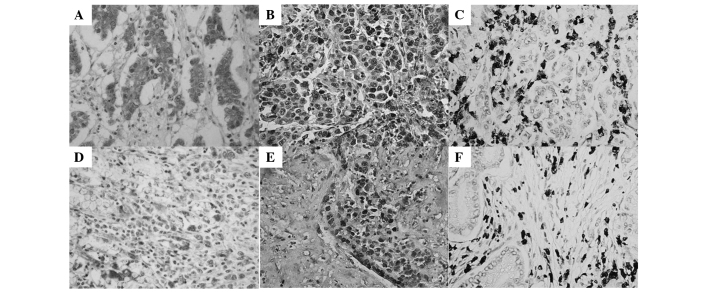
Expression of STAT3, p-STAT3 and HIF-1α in VM GAC and non-VM GAC tissues (hematoxylin and eosin stain; magnification, ×400). (A) STAT3, (B) p-STAT3 and (C) HIF-1α in VM GAC tissues. (D) STAT3, (E) p-STAT3 and (F) HIF-1α in non-VM GAC tissues. STAT3, signal transducer and activator of transcription-3; p-STAT3, phosphor-STAT3; HIF, hypoxia-inducible factor; VM, vasculogenic mimicry; GAC, gastric adenocarcinoma.

VM (Fig. 1A and B, arrow), characterized by CD31-negative/PAS-positive channels, and containing red blood cells, was only found in GAC specimens (31.7%; P<0.05). In the vascular wall of typical blood vessels from gastritis specimens, only CD31-positive/PAS-negative staining (Fig. 1C, arrow) was found instead of VM formation. STAT3-positive expression (Fig. 2A and D) was detected mainly in the cytoplasm and partly in the nuclei of GAC tissue cells. p-STAT3- (Fig. 2B and E) and HIF-1α-positive expression (Fig. 2Cand F) was detected mainly in the nuclei and partly in the cytoplasm of GAC tissue cells.

Positive expression levels of STAT3, p-STAT3 and HIF-1α were significantly increased in the GAC specimens compared with the gastritis specimens, respectively (81.7 vs. 15.0, 58.3 vs. 5.0 and 63.3 vs. 10.0%; P<0.05). Notably, STAT3-, p-STAT3- and HIF-1α-positive expression and VM formation in tissues from patients with lymph node metastasis were significantly higher than those from patients without lymph node metastasis, respectively (92.7 vs. 57.9, 75.6 vs. 21.1, 78.0 vs. 31.6 and 41.5 vs. 10.5%; P<0.05). In addition, STAT3- and p-STAT3-positive expression and VM formation were increased in poorly differentiated GAC tissues compared with those in well-differentiated GAC tissues, separately (94.1 vs. 65.4, 78.0 vs. 31.6 and 44.1 vs. 15.4%; P<0.05). The various expression levels of STAT3, p-STAT3 and HIF-1α were detected in VM GAC and non-VM GAC tissues, and it was found that STAT3 (Fig. 2A), p-STAT3 (Fig. 2B) and HIF-1α (Fig. 2C) showed higher expression, respectively, in VM GAC compared with non-VM GAC tissues (Fig. 2E, F and G) (P=0.012, P=0.013 and P=0.010, respectively). These results indicated a specific type of association between STAT3, p-STAT3, HIF-1α and VM formation (Table I).

### Correlation analysis of STAT3, p-STAT3, HIF-1α and VM in GAC tissues

The results showed that the expression levels of VM exhibited a positive correlation with those of STAT3 (r=0.480 and P=0.001), p-STAT3 (r=0.480 and P=0.001) and HIF-1α (r=0.480 and P=0.001), separately. The expression levels of HIF-1α were also positively associated with those of STAT3 (r=0.480 and P=0.001) and p-STAT3 (r=0.480 and P=0.001), separately (Table II).

### Survival analysis of STAT3, p-STAT3, HIF-1α and VM

**Figure 3:**
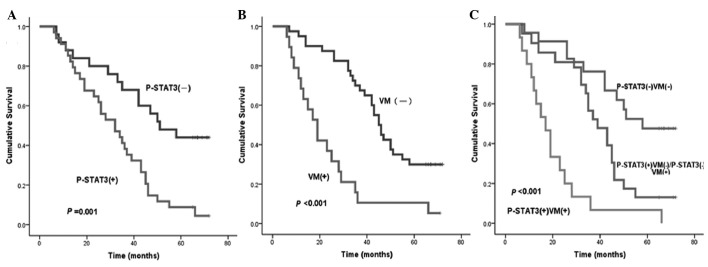
Survival curve in 60 GAC patients following curative resection of p-STAT3, VM and p-STAT3-VM. Negative and positive expression of (A) p-STAT3 and (B) VM. (C) Double-negative, single-positive and double-positive expression of p-STAT3 and VM. GAC, gastric adenocarcinoma; STAT3, signal transducer and activator of transcription-3; VM, vasculogenic mimicry; p-STAT3, phosphor-STAT3.

Using Kaplan-Meier univariate analysis, six factors were found to have statistically significant associations with the OS time of patients with GAC following curative surgery, including STAT3, p-STAT3 (Fig. 3A), HIF-1α, VM (Fig. 3B), status of lymph node metastasis and degree of differentiation (P<0.05). In addition, VM combined with STAT3, p-STAT3 or HIF-1α, respectively, was also found to have statistically significant associations with the OS time of patients with GAC. Patients with p-STAT3- and VM-negative expression were more likely to have a longer median OS time compared with those with p-STAT3- and (or) VM-positive expression (P<0.05) (Fig. 3C and Table III).

All the aforementioned six variables were analyzed by a multivariate Cox proportional hazards model (forward stepwise procedure). In this model, VM (HR, 3.021 and P=0.001), and p-STAT3 (HR, 2.520 and P=0.006) showed significant correlations with the OS times of patients with GAC following curative surgery, which indicated that VM and p-STAT3 were the independent risk factors of the OS time of patients with GAC (Table IV).

## Discussion

Recently, various factors have been studied to reveal the mechanism of VM formation. VM is considered to play a key role in tumor growth, progression and metastasis (5,6). Li *et al* (5) found the existence of VM in GAC, and that hypoxia may participate in VM formation of GAC, particularly in poorly differentiated GAC. In the present study, it was found that VM was detected only in GAC specimens, particularly in the poorly differentiated GAC tissues. Patients with VM formation had a significantly shorter median OS time than those without VM formation (P<0.001). By multivariate survival analysis, VM was found to be an independent risk factor of the OS time of patients with GAC. Therefore, VM was indicated to be a detective marker of GAC tissues.

The most significant difference in the microenvironment between tumor and normal tissues is ischemia of the tumor due to structural imperfections of the tumor vessels, which induces to anoxia of tumor tissues (11). As a hypoxia-dependent protein, HIF-1α can be rapidly degraded when oxygen is normal, but when oxygen is not sufficient, it can upregulate cell proliferation at the transcription level, activate the expression of numerous hypoxia response genes, and be closely associated with energy metabolism, angiogenesis, infiltration and metastasis of the tumor by binding with the hypoxia response element of the hypoxia response (12). As a tumorigenesis factor, HIF-1α could induce angiogenesis of lung cancer when activated in hypoxia (13). In the present study, it was found that HIF-1α-positive expression was significantly increased in GAC specimens, particularly in VM GAC specimens, compared with the gastritis specimens (P<0.05). Similarly, the HIF-1α-positive expression was positively associated with VM formation (r=0.480 and P=0.001). These demonstrated that HIF-1α was a positive index of VM formation in GAC tissues. Patients with HIF-1α- and VM-positive expression were more likely to have a shorter median of OS compared with those with HIF-1α- and (or) VM-negative expression by survival analysis (P<0.05). Therefore, we propose that the phenomenon of HIF-1α-VM double-positive expression is a more promising index of prognosis than that of HIF-1α- or VM-positive expression.

As a member of the STAT family, STAT3 plays a significantly important role in human cancers, and is closely associated with the proliferation and apoptosis of tumor cells in a wide variety of tumor types. A study by Yakata *et al* (14) showed increased expression of STAT3 in gastric cancer, and found that STAT3 expression was significantly associated with invasion depth and lymph node metastasis of GAC tissues. STAT3 could be transformed into p-STAT3 by activation under hypoxic conditions. In the present study, the expression levels of STAT3 and p-STAT3 were found to be higher in GAC than those in gastritis tissues, particularly in poorly differentiated GAC (P<0.05). This result agreed with the findings of Yakata *et al* (14).

Xu *et al* (15) demonstrated that HIF-1 expression induced by Src was inhibited when blocking STAT3 signaling in breast cancer and melanoma cell lines. STAT3 converted to p-STAT3, and p-STAT3 directly bound HIF-1α and upregulated HIF-1α stability through delaying protein degradation and accelerating protein synthesis (7). STAT3 can promote HIF-1α transcription and increase HIF-1α protein stability by inhibiting the expression of p53 (8,16,17). All these results reveal that STAT3 is a positive factor of HIF-1α. Furthermore, p-STAT3 can upregulate the expression of matrix metalloproteinase 2 (MMP2) to promote the formation of VM in tumor tissues (6,18–20). Hypoxia is a possible mechanism of VM genesis by the induction of the expression of HIF-1α, MMP-2 and MMP-9 (6,19,20). Above all, the results of the present study concluded that STAT3 activation could upregulate and stabilize the expression of HIF-1α by various pathways intending to promote the VM formation under hypoxic conditions.

The results of the present study showed that STAT3- and p-STAT3-positive expression was increased in the VM group (P<0.05). Additionally, STAT3 expression was positively correlated with p-STAT3, HIF-1α and VM expression, respectively, in GAC tissues. By univariate and multivariate survival analysis, patients with both negative expression of p-STAT3 and VM were found to be more likely to have a longer median OS time compared with those with p-STAT3- and (or) VM-positive expression (P<0.05), and p-STAT3 was an independent risk factor of the OS time of patients with GAC. These indicated a specific type of association between STAT3, p-STAT3, HIF-1α and VM in GAC tissues.

Combining the aforementioned studies with the results of the present study, it was deemed that STAT3 may be a novel positive factor of VM formation in GAC tissues through the effect of p-STAT3. STAT3 and p-STAT3 were positive factors of HIF-1α expression and VM formation in GAC tissues. STAT3 was significantly associated with progression and prognosis of GAC. Combining the previous studies with the present study results, it can be concluded that STAT3 may promote the formation of VM to affect the invasion and metastasis in GAC tissue by a specific type of mechanism (STAT3-p-STAT3-HIF-1α-VM).

In conclusion, it was found that p-STAT3 and VM played a significant role in indicating the prognosis of patients with GAC. STAT3 activation may play a positive role in VM formation of GAC tissues by the STAT3-p-STAT3-HIF-1α-VM effect axis. These results provide opportunities to develop potential novel therapeutic targets for GAC.

## Figures and Tables

**Table I:** Correlation between STAT3, p-STAT3, HIF-1α, VM and clinicopathological parameters

	STAT3			p-STAT3			HIF-1α		
			
Factors	Positive	Negative	P value	Positive	Negative	P value	Positive	Negative	P value
Group									
Gastritis	3	17	<0.001	1	19	<0.001	2	18	<0.001
GAC	49	11		35	25		38	22	
Gender									
Male	37	9	0.958^a^	25	21	0.256	28	18	0.473
Female	12	2		10	4		10	4	
Age at surgery, years									
<60	19	8	0.087^a^	15	12	0.693	15	12	0.258
≥60	30	3		20	13		23	10	
Tumor size, cm									
<5	20	6	0.406	14	12	0.538	14	12	0.182
≥5	29	5		21	13		24	10	
Status of lymph node metastasis									
0	11	8	0.007^b^	4	15	<0.001	6	13	0.002
1–6	25	2		21	6		20	7	
>6	13	1		10	14		12	2	
Degree of differentiation									
Poor	32	2	0.012^a^	25	9	0.006	24	10	0.182
Mid to well	17	9		10	16		14	12	
TNM stage									
I-II	21	5	0.875	15	11	0.930	13	13	0.061
III-IV	28	6		20	14		25	9	
VM									
Positive	19	0	0.032^a^	16	3	0.006	17	2	0.004
Negative	30	11		19	22		21	20	

a χ^2^ test of continuous correction;

b Fisher's exact test.

STAT3, signal transducer and activator of transcription-3; p-STAT3, phosphor-STAT3; HIF-1α, hypoxia-inducible factor-1α; GAC, gastric adenocarcinoma; TNM, tumor-node-metastasis; VM, vasculogenic mimicry.

**Table II:** Correlation between STAT3, p-STAT3, HIF-1α and VM expression in GAC.

	STAT3				p-STAT3				HIF-1α			
			
Factors	Negative	Positive	P value	r	Negative	Positive	P value	r	Negative	Positive	P value	r
VM												
Negative	11	30	0.012	0.323	22	19	0.028	0.285	20	21	0.004	0.369
Positive	0	19			3	16			2	17		
HIF-1α												
Negative	9	13	<0.001	0.444	14	8	0.008	0.339				
Positive	2	36			11	27						
p-STAT3												
Negative	9	16	0.002	0.386								
Positive	2	33										

STAT3, signal transducer and activator of transcription-3; p-STAT3, phosphor-STAT3; HIF-1α, hypoxia-inducible factor-1α; VM, vasculogenic mimicry; GAC, gastric adenocarcinoma.

**Table III:** Univariate analysis of factors affecting the overall survival time of 60 patients with GAC by the Kaplan-Meier method.

Factor	χ^2^	P-value
Gender	0.001	0.976
Age	0.449	0.503
Tumor size	1.664	0.197
Depth of primary tumor invasion	0.220	0.639
Status of lymph nodes metastasis	9.312	0.002
Degree of differentiation	5.506	0.019
TNM stage	1.374	0.241
STAT3	9.271	0.002
p-STAT3	11.793	0.001
HIF-1α	8.013	0.005
VM	18.312	<0.001
VM and STAT3	16.301	<0.001
VM and p-STAT3	29.102	<0.001
VM and HIF-1α	26.305	<0.001

GAC, gastric adenocarcinoma; TNM, tumor-node-metastasis; STAT3, signal transducer and activator of transcription-3; p-STAT3, phosphor-STAT3; HIF-1α, hypoxia-inducible factor-1α; VM, vasculogenic mimicry.

**Table IV:** Multivariate analysis of factors affecting the overall survival time of patients with GAC by Cox proportional hazards model.

Factor	P-value	Relative risk (HR)	95%CI
Status of lymph node metastasis	0.100		
Degree of differentiation	0.364		
STAT3	0.164		
p-STAT3	0.006	2.520	1.310–4.849
HIF-1α	0.244		
VM	0.001	3.021	1.613–5.660

GAC, gastric adenocarcinoma; HR, hazard ratio; CI, confidence interval; STAT3, signal transducer and activator of transcription-3; p-STAT3, phosphor-STAT3; HIF-1α, hypoxia-inducible factor-1α; VM, vasculogenic mimicry.

## References

[1] Maniotis AJ, Folberg R, Hess A (1999). Vascular channel formation by human melanoma cells in vivo and vitro: vasculogenic mimicry. Am J Pathol.

[2] Liu WB, Xu GL, Jia WD (2011). Prognostic significance and mechanisms of patterned matrix vasculogenic mimicry in hepatocellular carcinoma. Med Oncol.

[3] Sun W, Shen ZY, Zhang H (2012). Overexpression of HIF-1α in primary gallbladder carcinoma and its relation to vasculogenic mimicry and unfavourable prognosis. Oncol Rep.

[4] Wu S, Cheng Z, Yu L, Song W, Tao Y (2011). Expression of CD82/KAI1 and HIF-1α in non-small cell lung cancer and their relationship to vasculogenic mimicry. Zhongguo Fei Ai Za Zhi.

[5] Li M, Gu Y, Zhang Z (2010). Vasculogenic mimicry: a new prognostic sign of gastric adenocarcinoma. Pathol Oncol Res.

[6] Sun B, Qie S, Zhang S (2008). Role and mechanism of vasculogenic mimicry in gastrointestinal stromal tumors. Hum Pathol.

[7] Jung JE, Lee HG, Cho IH (2005). STAT3 is a potential modulator of HIF-1-mediated VEGF expression in human renal carcinoma cells. FASEB J.

[8] Pawlus MR, Wang L, Murakami A, Dai G, Hu CJ (2013). STAT3 or USF2 contributes to HIF target gene specificity. PLoS One.

[9] Chen QR, Guan F, Yan DJ, Lei DS, Fu L (2012). The dynamic expression of allograft inflammatory factor-1 in hepatic tissues and splenic cells of BALB/c mice with *Schistosoma japonicum* infection. Tissue Antigens.

[10] Yu HF, Zhao G, Ge ZJ (2012). High RIN1 expression is associated with poor prognosis in patients with gastric adenocarcinoma. Tumour Biol.

[11] Crowther M, Brown NJ, Bishop ET, Lewis CE (2001). Microenvironmental influence on macrophage regulation of angiogenesis in wounds and malignant tumors. J Leukoc Biol.

[12] Huang GW, Yang LY, Lu WQ (2005). Expression of hypoxia-inducible factor 1 alpha and vascular endothelial growth factor in hepatocellular carcinoma: Impact on neovascularization and survival. World J Gastroenterol.

[13] Noman MZ, Buart S, Van Pelt J (2009). The cooperative induction of hypoxia-inducible factor-1 alpha and STAT3 during hypoxia induced an impairment of tumor susceptibility to CTL-mediated cell. J Immunol.

[14] Yakata Y, Nakayama T, Yoshizaki A (2007). Expression of p-STAT3 in human gastric carcinoma: significant correlation in tumour invasion and prognosis. Int J Oncol.

[15] Xu Q, Briggs J, Park S (2005). Targeting Stat3 blocks both HIF-1 and VEGF expression induced by multiple oncogenic growth signaling pathways. Oncogene.

[16] Hu CJ, Wang LY, Chodosh LA, Keith B, Simon MC (2004). Differential roles of hypoxia-inducible factor 1alpha (HIF-1alpha) and HIF-2alpha in hypoxic gene regulation. Mol Cell Biol.

[17] Niu G, Wright KL, Ma Y (2005). Role of Stat3 in regulating p53 expression and function. Mol Cell Biol.

[18] Xie TX, Wei D, Liu M (2004). Stat3 activation regulates the expression of matrix metalloproteinase-2 and tumor invasion and metastasis. Oncogene.

[19] Sun B, Zhang D, Zhang S, Zhang W, Guo H, Zhao X (2007). Hypoxia influences vasculogenic mimicry channel formation and tumor invasion-related protein expression in melanoma. Cancer Lett.

[20] Xu X, Jia R, Zhou Y, Song X, Fan X (2010). Investigation of vasculogenic mimicry in sebaceous carcinoma of the eyelid. Acta Ophthalmol.

